# Perspectives of Individuals With Obsessive-Compulsive Disorder on the Role of Artificial Intelligence in Therapy and Treatment: Thematic Qualitative Study

**DOI:** 10.2196/98822

**Published:** 2026-07-31

**Authors:** Daniel Mokhtar, Harrison Wang, Emma C Garland, Dejan Shakya, Lucas Occhino-Moede, Xiao Liu, Adam Charles Frank

**Affiliations:** 1 Department of Psychiatry and Behavioral Sciences, Keck School of Medicine, University of Southern California, 2500 Alcazar St, Suite 2200, Los Angeles, CA, 90033, United States, 1 323-442-6000, 1 323-442-6001

**Keywords:** artificial intelligence, AI, large language model, LLM, chatbot, OCD, obsessive-compulsive disorder, patient experience, qualitative, thematic analysis

## Abstract

**Background:**

AI has become increasingly used in mental health care for applications such as diagnosis, monitoring, and treatment support. These include tools like clinician support systems, large language models, and conversational agents used to augment psychotherapy and clinical decision-making. While prior research suggests potential benefits of and concerns with AI, little is known within the domain of obsessive-compulsive disorder (OCD). Given the expanding role of AI in psychiatry, understanding these perspectives is essential to ensuring AI implementation aligns with patient priorities and values.

**Objective:**

This study aims to explore the perspectives of individuals with OCD on the use of AI in health care, including perceived benefits, risks, and its role in relation to human clinicians.

**Methods:**

We conducted semistructured interviews with 24 adults self-reporting OCD, recruited through online communities and advocacy networks. Eligible individuals (≥18 y with self-reported OCD) completed screening, provided informed consent, and participated in remote Health Insurance Portability and Accountability Act (HIPAA)-compliant Zoom (Zoom Communications, Inc) interviews (May-December 2024). Transcripts were deidentified, open-coded, and used to develop a codebook. Focused codes were applied using a thematic analysis framework in Dedoose (v9.2.22; Sociocultural Research Consultants, LLC). Each transcript was independently coded by 2 reviewers, with discrepancies resolved through consensus. Themes were developed through iterative interpretive analysis of code clusters.

**Results:**

Participants’ perspectives encompassed concerns and benefits of AI in mental health care. Participants expressed concerns about the accuracy and efficacy of information provided by AI, as well as a limited ability for clinical judgment in psychiatric care. Additionally, participants emphasized the importance of human connection, particularly therapeutic alliance, empathy, and reassurance provided by clinicians, which they felt AI could not replicate. Concerns about data privacy, security, and downstream use of information were also highlighted. Despite concerns, many endorsed the use of AI as an adjunct rather than a replacement for clinicians, noting potential benefits in symptom monitoring, preliminary information gathering, and support for administrative tasks, provided that human oversight is maintained.

**Conclusions:**

Individuals with OCD expressed nuanced views on AI in mental health care, balancing cautious optimism with several concerns. While AI may improve efficiency, standardization, and symptom monitoring, participants highlighted risks related to deindividualization, accuracy, and erosion of human connection. These findings underscore the importance of patient-centered, ethically guided AI integration that preserves the therapeutic alliance while leveraging technological benefits.

## Introduction

Obsessive-compulsive disorder (OCD) is a chronic and often disabling psychiatric condition characterized by intrusive thoughts and repetitive behaviors that cause significant distress and functional impairment across occupational, academic, and interpersonal domains [1,2]. Lifetime prevalence estimates range from 1% to 3%, and patients often experience a waxing and waning of symptoms over time [3,4]. Modalities such as psychotherapy with exposure and response prevention (ERP) and pharmacotherapy with selective serotonin reuptake inhibitors are evidence-based and can produce meaningful symptom reduction [5]. Unfortunately, despite the existence of effective treatments, many individuals with OCD do not receive timely or adequate care [6].

Given the chronicity of OCD and gaps in access to evidence-based care, there is increasing interest in augmenting current treatment models with AI. AI has evolved from a largely theoretical concept in the mid-20th century into a rapidly advancing field with broad implications for health care delivery [7]. Improvements in computational power, widespread digitization of health information, and advances in algorithmic design have enabled AI systems capable of identifying complex patterns in large datasets [8], with applications including pattern recognition, prediction, and decision-making [9]. In medicine and mental health care settings, this includes supporting diagnosis, monitoring, and intervention. For instance, machine learning (ML) algorithms demonstrate promising accuracy in detecting psychiatric conditions and predicting outcomes [10]. AI-enabled clinical decision support systems have been developed that assist with medication selection and adjustment. Indeed, an AI-clinical decision support system used in the treatment of moderate major depressive disorder supported improvements in response and remission rates [11].

AI-driven conversational agents and chatbots have been used to augment psychotherapy by delivering components of cognitive behavioral therapy, providing psychoeducation, and offering real-time coping support [12]. Evidence indicates, particularly for adolescents and young adults, these tools can reduce depressive, anxiety, stress, and psychosomatic symptoms [13,14]. Such systems are often positioned as scalable adjuncts to therapy, extending support between sessions or to individuals without access to in-person care.

The successful integration of AI depends not only on technical performance but also on patient trust, acceptability, and lived experience. Understanding how patients perceive and interact with AI-enabled tools is critical to ensure these innovations promote equitable access, improve outcomes, and avoid unintended harms. Although research has begun to explore attitudes toward AI in mental health care, it remains limited and largely condition-agnostic. Survey studies indicate perceived benefits, such as improved access and efficiency, alongside concerns about privacy, accuracy, bias, and effects on the therapeutic relationship [15]. Research on digital mental health technologies more generally suggests that beliefs about credibility, personalization, and emotional understanding influence engagement [15]. However, little is known about how individuals with OCD perceive AI-enabled interventions, or how OCD symptomatology may shape these perceptions.

Given persistent treatment gaps and the growing role of AI in psychiatric care, condition-specific, patient-centered research is needed. Within a 2025 sample of 499 US adults with self-identified mental health conditions, nearly half reported using chatbots for psychological support last year [16]. Core features of OCD, such as intolerance of uncertainty and compulsive reassurance seeking, may uniquely predispose individuals with OCD toward maladaptive AI use [17]. Understanding how patients with OCD conceptualize these dynamics is a necessary precursor to designing safe and effective AI tools for this population. We conducted a qualitative study using a thematic analysis framework to explore how individuals with OCD perceive the use of AI in treatment, including benefits, risks, and conditions for acceptability. Given the existing literature described above and our clinical experience, we postulated that adults with OCD would have similarly varying perspectives on technology.

## Methods

### Study Design

This study sought to explore the attitudes of people with OCD toward the use of AI in their health care. We then used the approach of thematic analysis to interpret our data [18,19]. We used the COREQ (Consolidated Criteria for Reporting Qualitative Research) framework, a validated checklist for qualitative reporting, to guide and document important aspects of the research team, methodology, findings, and analysis (Checklist 1) [20].

### Recruitment and Sample

Recruitment processes and participants were identical to Occhino-Moede et al [15]. Participants were recruited via convenience sampling from May to December 2024 through online spaces focused on OCD education, advocacy, and peer support, including platforms affiliated with the International OCD Foundation and OCD research registries maintained by our laboratory.

Recruitment posts described the study purpose, eligibility criteria, participation requirements, and compensation. Interested individuals contacted the research team and completed a REDCap (Vanderbilt University) screening survey. Eligible respondents were invited to a virtual onboarding session to verify submitted answers, review procedures, and provide informed consent.

Eligibility criteria included self-reported age ≥18 years and a diagnosis of OCD. Twenty-seven participants completed consent and an interview. Interviews were completed by personnel trained in OCD symptomatology; 3 were excluded due to concerns about eligibility (eg, unclear diagnosis or suspected repeat participation). Camera-on interviews and brief discussion of OCD symptoms were used to mitigate this risk. No participants withdrew after enrollment. Seven participants had prior contact with laboratory personnel through other studies. No repeat interviews were conducted. Sample characteristics are summarized in Table 1.

**Table 1.** Participant demographics (N=24).

Characteristics	Values
Median age, years (range, IQR)	26.2 (20.1‐63.6, 12.8)
Gender	
Man	7
Woman	16
Nonbinary	1
State	
California	14
Pennsylvania	3
Illinois	2
Ohio	2
Florida	1
Maryland	1
Virginia	1

### Research Team and Procedures

The study was conducted by a multidisciplinary team, with formal educational levels ranging from undergraduate to medical student to board-certified psychiatrist; the team was led by the principal investigator (PI; ACF, MD/PhD, psychiatrist) (Multimedia Appendix 1). The PI has experience working with individuals with OCD in an outpatient clinical setting providing psychotherapy, medication management, and neuromodulation; additionally, the PI conducts quantitative and qualitative research in other studies with adults with OCD. While the interview guide did not exclusively focus on clinical or treatment-related applications of AI, given the PI’s clinical background, these topics may be emphasized. All interviewers completed a structured 6-week qualitative training (eg, review of the interview guide, observation of senior interviewers, and postinterview debriefings) and participated in ongoing supervision. Interviewers were also trained in OCD symptomatology by the study PI; this included review of *Diagnostic and Statistical Manual of Mental Disorders, 5th Edition* (*DSM-5*) [21] criteria for OCD and review of diagnostic assessments used for OCD including the Yale Brown Obsessive Compulsive Scale (YBOCS) [22] and YBOCS symptom checklist, so that interviewers understood the breadth of experiences and symptoms that can be manifest in OCD. Some interviewers identified as having lived experience with OCD; others did not. All interviewers were familiar with commonly available forms of AI when interviews were completed, such as large language models (LLMs), chatbots, and text-to-image generating services. Some interviewers identified as consistent users of these AI services, and some identified as occasional users.

We used a semistructured interview guide to elicit participants’ experiences with digital technologies used for health and health care, with a focus on OCD-related care. Participants were reminded of the study goals, and interviewers introduced themselves and their role in the project. Questions explored when and how participants began using various technologies, how these tools fit into their daily routines, perceived benefits and drawbacks, and how they related these tools to OCD symptoms, help-seeking, and treatment. This interview guide was used in Occhino-Moede et al [15], and while it included one specific question about AI, participant-initiated discussions about AI occurred outside of the context of this single question. A sample of the interview guide is provided in Multimedia Appendix 2.

Interviews were conducted remotely via Health Insurance Portability and Accountability Act (HIPAA)-compliant Zoom (Zoom Communications, Inc) between May 16, 2024, and December 20, 2024. With participant permission, sessions were audio- and video-recorded and auto-transcribed within Zoom. Research assistants reviewed transcripts for accuracy and deidentification. Interviews ranged from 35 to 65 minutes, with a median of 51 minutes.

### Data Coding

Given the breadth of discussion of AI that occurred in interviews conducted in Occhino-Moede et al [15], we developed a new codebook for this manuscript. Codebook development was informed by Braun and Clarke’s [18] 6-phase framework for thematic analysis. Two members of the research team (LO and EL) independently conducted open coding of the first five transcripts to generate an initial set of inductive codes related to participants’ perspectives on AI. Analytic memos were maintained during this process to capture emerging interpretations and support reflexivity. The coders then met to compare codes and memos and used constant comparison to consolidate conceptually similar codes into broader candidate themes. Codes and their definitions were iteratively refined against the data set, and hierarchical relationships between parent and child codes were established. This recursive process produced the final codebook, which included global codes and nested thematic categories reflecting patterns identified in participant narratives. An excerpt of the final codebook is provided in Multimedia Appendix 3.

All transcripts were imported into Dedoose, and the codebook was implemented in the software. Each interview was coded by two team members working independently. During coding, researchers identified and coded all AI-related content across the interview transcripts, including both responses to AI-specific interview questions and spontaneous references to AI that emerged during broader discussions of technology use in health care. Coder pairs subsequently met to reconcile differences through consensus.

During consensus, team members discussed whether any segments of text relevant to the research question had not been adequately captured by existing codes. These novel concepts were added to the existing codebook, and all previous transcripts were re-coded using the revised structure. Coding continued until code saturation was reached, defined as the point at which no new codes emerged from the data—reflecting the range of thematic issues identified across interviews [23].

### Theory Development

Following consensus coding using the finalized codebook, the analytic team shifted from descriptive coding to interpretive theme development. Guided by the 4R’s framework described by Naeem et al [24], researchers examined how codes combined to reflect broader patterns in participants’ perspectives on AI in health care.

First, coded excerpts were reviewed across transcripts to ensure codes accurately reflected the underlying data and were applied consistently. The team then reflected on relationships among codes, using analytic memos and group discussion to explore how clusters of codes captured shared meanings within participants’ narratives.

Candidate themes were subsequently refined through iterative team meetings in which codes were grouped into broader conceptual categories and evaluated for coherence and distinctiveness. Discordant cases were reviewed to ensure themes captured variation within the dataset. Negative cases are noted in the results, as these occurred in some themes [25]. Final themes were defined and named to represent the central patterns identified in participant narratives, with representative excerpts selected to illustrate each theme.

### Ethical Considerations

All study procedures were approved by the University of Southern California Institutional Review Board (UP-23‐01094). All study procedures involving human participants adhered to the ethical standards of the institutional review board and the 1964 Declaration of Helsinki and its subsequent amendments. Prospective participants received written information describing the study purpose, procedures, risks, benefits, and voluntary nature of participation. Study staff reviewed this information during a videoconference consent visit and answered questions prior to obtaining informed consent. Participants could decline any question or withdraw at any time without penalty. Interview data were deidentified before analysis and stored on secure, access-restricted servers. Participants received a US $50 Tango gift card after completing the interview.

### Participants

A description of the participants (n=24) is provided in Table 1. No participants refused to participate in the study or withdrew their participation.

## Results

### Theme A: Inaccuracy and Inefficacy of AI in Health Care

Participants expressed concern that AI might generate inaccurate, fabricated, or misleading information. These inaccuracies, participants noted, could potentially lead to users feeling misunderstood at best, or at worst, engaging in dangerous behaviors.

Participants expressed specific concerns about AI’s accuracy, such as worries about misattributed source information or LLM hallucinations, which are an established phenomenon in which LLMs identify nonexistent patterns, resulting in outputs that are incorrect or nonsensical [20]:

The degree to which it is erroneous in what it is saying is frustrating when it’s a topic I know about, and have engaged with it. It’s frustrating, again, because you can’t see through that unless you know already. Like, it’ll present it as though, like ‘this is the quotation you're looking for’, and you’re like, no, that quotation is not from that.

[Participant 16]

AI produces what’s called hallucinations, where it makes up stuff. And I’ve also experienced that when I’ve been using it personally. That it would say something that I knew was not correct. That’s a recognized problem with AI.

[Participant 3]

With regard to OCD treatment, participants expressed concerns about AI’s ability to understand the individual nature of the disorder and provide effective treatment recommendations, citing examples of how well-intentioned but misguided care can negatively affect outcomes:

Everyone has different obsessions and compulsions, so like, I think there would just have to be a lot that would have to like go into that before it (AI) could be like really, like effective and not harmful in the mental field in particular.

[Participant 19]

Treating it (OCD) as just kind of like anxiety … just makes it a lot worse. So even though it’s not like someone acting in a harmful way, if it’s not the correct treatment or thing to do … if someone is left feeling like they weren’t helped, then that can just make it a lot worse and more isolating for them.

[Participant 19]

Some participants described concerns about the efficacy of AI in the context of mental health care more broadly. One individual stated:

I remember like, several months ago, reading something about AI, with either eating disorders, or a suicide hotline type of situation where it was like wildly ineffective… there’s just like a lot of kinks there to be worked out like if it were to ever be used for … helping people with … mental health concerns.

[Participant 19]

While a few participants were highly doubtful of the accuracy and efficacy of AI in treatment, most expressed feeling that they might be comfortable in the future, but didn’t feel that the technology was quite developed enough yet. One participant succinctly stated:

I don’t think that AI is quite there where it’s like 100% trustworthy, and there’s different mistakes that it can make.

[Participant 3]

However, not every participant shared this sentiment. When asked whether they had any concerns about AI and treatment, one participant acknowledged that mistakes were possible, but considered them unlikely:

Just giving the wrong diagnosis and just mak[ing] things worse. But I think that at the level where we are at, I think it shouldn’t be an issue.

[Participant 19]

Concerns about LLM hallucinations, erroneous source information, weak algorithms, or poor quality, and even potentially dangerous outputs, decrease confidence in the ability of AI to generate helpful and accurate information for users with OCD.

### Theme B: Clinical Decision-Making as a Contextual, Integrative, and Experiential Process

Participants emphasized the importance of clinical judgment in psychiatric care, describing it as a process grounded in contextual, interpretative, and experiential reasoning. Flexibility, nuance, and professional training were identified as central components, suggesting that effective psychiatric care depends on the ability to interpret complex and often ambiguous clinical presentations.

Participants described one component of effective psychiatric care as the ability of the clinician to detect subtle cues that extend beyond explicit symptom reporting. Clinical judgment was framed as requiring attention to tone, affect, and contextual signals that may not be directly verbalized. One participant noted:

A lot of psychiatry is more than just what the patient is saying… it’s observations, listening, how they’re saying it, trying to understand the bigger picture of their life, you know, what factors outside of what they’re just talking about.

[Participant 9]

When asked if they had positive experiences with clinicians which would have been different with AI, one patient responded:

Oh my gosh, yeah, 100%... [my provider] looked holistically at my whole life story, and could understand what could be a trauma response, what could be a personal thing… sometimes you don’t necessarily have the verbiage exactly to express, like what’s going on, especially when you’re new to the field of psychiatry.

[Participant 14]

Beyond recognizing cues, participants emphasized that psychiatric decision-making should be contextual and integrative, rather than linear and formulaic. Rather than relying on isolated symptoms, clinicians were described as integrating life history, social determinants, and situational factors. One participant commented that:

There’s a reason, you know, it’s not robots doing [medication adjustment or therapy]… with diagnosing and knowing the individual and the situation and the social determinants that are affecting it, there’s more than just like the right answer.

[Participant 9]

Clinical reasoning was also described as requiring adaptability rather than rigid adherence to standardized patterns. One participant described their experience with rare medication side effects:

I’m that point less than point 1%. I have had a super rare side effect to everything that I’ve taken so I need to be able to interact with an actual human when adjusting things. If we mess something up or go up too high, I’ll have a weird side effect. So I definitely would want a human that knows that and isn’t just a computer and going off of a formula.

[Participant 24]

Their concerns suggest that participants viewed good clinical judgment as flexible and individualized, particularly when patients’ experiences fell outside the norm.

Finally, participants linked legitimate clinical decision-making to formal training and credentialing. Decisions such as prescribing medication or modifying treatment plans were viewed as requiring education and supervised experience, not merely access to data. One participant stated:

A doctor, they go through so much training and education… they really possess the knowledge that is required to give the medication or prescription. The AI doesn’t.

[Participant 12]

Although these perspectives predominated, one participant suggested that certain components of clinical reasoning, namely preliminary information gathering, could be appropriately delegated to AI. As they explained:

Being honest, I think AI will be helpful to some certain extent, like, let’s say, the initial analysis… The AI can categorize me into some specific category of patient or something. And then, based on that, the doctors or anyone can come in after the initial analysis is done

[Participant 10]

Though they clearly saw the utility of AI in health care systems, it was important that clinicians retained responsibility for interpretation and final decision-making. This perspective suggests that participants distinguished between routine, structured aspects of clinical reasoning and the more complex interpretive processes they viewed as uniquely human.

Overall, participants distinguished human clinical reasoning from AI-generated outputs by emphasizing cue recognition, contextual interpretation, flexible clinical judgment, and credentialed expertise. In their accounts, effective psychiatric care was closely tied to these forms of clinical reasoning and difficult to replicate through automated systems.

### Theme C: Therapeutic Alliance, Human Connection, and Trust

Participants emphasized that human connection is central to effective OCD care. Across interviews, trust, reassurance, relational nuance, and individualized understanding were described as important to symptom management and therapeutic progress. While part of this therapeutic alliance was observed to be a result of the providers’ expertise and training as described in Theme B, another key theme emerged which displayed the importance of the human connection in itself.

Many participants described the human connection as uniquely reassuring, particularly during periods of symptom exacerbation. One participant reflected:

I found it very reassuring when I’m having an OCD flare to talk to someone who I know is an expert on this subject and he’s treated people before who can say ‘No, this doesn’t mean that you're insane’...That can be very reassuring

[Participant 3]

Similar to Theme B, this participant also viewed clinical experience as important; however, the reassurance it provided was grounded not only in expertise, but in the relational presence of a clinician who could validate fears in real time.

The relational alliance between patients and providers was described as essential by many participants. This perspective framed therapy not merely as information exchange, but as an interpersonal process grounded in empathy. For instance, therapists who made a personal connection or shared an anecdote were seen as uniquely helpful. As one participant explained:

Also, I found it very reassuring or helpful when a therapist will help customize a therapeutic technique to my OCD. Or even I’ve had a therapist in the past who related stories about themselves in their battle with obsessive thoughts

[Participant 3]

Another stated:

Well, a therapist, I think, is meant to soothe you, listen to you individually and soothe you… You can’t take away from the human aspect of a therapist.

[Participant 11]

AI was frequently positioned as incapable of replicating these relational dimensions. Therapeutic care was often viewed as part of broader human connectedness and community belonging. One participant expressed discomfort with replacing human interaction, stating:

It’s just really icky to me that we’re replacing these human interactions through AI…we need to feel part of the community…we’re functioning with humans hopefully, so I feel like it’s just not good to be kind of withdrawn from humanity.

[Participant 14]

Overall, participants framed therapeutic alliance, characterized by reassurance, personalization, empathy, and human touch, as indispensable to effective OCD care and not readily replaceable by AI systems.

### Theme D: Data, Surveillance, Privacy, and Emotional Vulnerability

Participants described concerns regarding privacy, data security, downstream uses of personal data, and emotional vulnerability when considering the use of AI in health care. A central tension emerged between trust in human clinicians and uncertainty about whether AI systems could uphold comparable standards of confidentiality and privacy.

Participants often raised concerns regarding privacy and security of sensitive information broadly. Several participants explicitly contrasted their confidence in health care providers with their hesitancy toward AI systems, expressing concern about the possibility of data breaches. One participant stated:

I totally trust health care providers to, you know, uphold HIPAA and um like health privacy, but the moment that AI is used- I don’t know- I don’t know how I can trust AI to maintain that same level of health security as like a doctor

[Participant 1]

Another participant explicitly stated:

I guess security is always a concern…data stealing from the therapy session would be a concern.

[Participant 3]

Participants also expressed concern about losing autonomy over their personal information once it is collected or processed by AI systems. One participant described involvement in a research study using AI and questioned how their recorded data would be handled:

I was doing another or currently am doing another research study where they’re doing AI as well. And it’s something about low back pain. So like I had to be recorded while I was doing different exercises and they said it was AI stuff and I was like, well, what, what are they going to do with this?

[Participant 20]

Another participant voiced fears of misuse and impersonation, stating:

I’m concerned that it, you know, that it’s going to be abused…It’s scary that way. I mean, it’s like somebody could pretend to be me

[Participant 23]

Beyond technical concerns, participants distinguished emotional vulnerability as a separate and equally important issue. One participant highlighted discomfort with emotional disclosure to nonhuman systems:

Safety also, intimacy also. Someone might not be really comfortable in sharing these things with a Chatbot or any AI engine, because you never know how your data might get used or how it is interpreted.

[Participant 10]

Together, these accounts illustrate how concerns about AI in health care extend beyond technical data security to include autonomy, trust, emotional comfort, and the broader psychological implications of sharing sensitive information with artificial systems.

### Theme E: Participant Supported Uses of AI in Health Care

Participants generally endorsed the use of AI as a supportive clinical tool when implemented transparently, complementarily, and under human supervision. Rather than rejecting AI outright, many described conditional acceptance, particularly for functions that improve efficiency, organization, and symptom monitoring, as long as clinicians retain interpretive authority.

Several participants consistently emphasized that AI should assist rather than replace clinicians. However, one participant provided an example of potential dislike for AI that stood out from other cases:

I know that if it was AI that was helping to understand or confirm the evidence-based treatment, I would have no problem with that, as I would with the physician or therapists looking up information from any source. But I would dislike if the AI was answering me directly instead of the therapist.

[Participant 3]

Participants also described AI as potentially useful for initial analysis and administrative tasks, recognizing improvements in efficiency and standardization without supplanting clinician involvement. One participant explained:

I think AI will be helpful to some certain extent…The AI can categorize me into some specific category of patient…based on that, the doctors or anyone can come in after the initial analysis is done, so I feel that will save time as well.

[Participant 10]

When asked about potential positive uses of AI in health care, the same participant elaborated on the value of streamlining intake processes:

Especially…for therapy, or something like in the initial sessions, [with] the basic general questions…Those can be done via some, let’s say, form…then it can be boiled down to something.

[Participant 10]

Participants also identified potential applications for AI in supporting evidence-based therapeutic interventions. One participant suggested that AI could assist with the development of ERP exercises while still operating within a structured clinical framework:

AI could probably be used well to kind of come up with ERP scripts and, like easy ready to go ERP scripts…AI with ERP would probably work really well…with ERP specifically, like, it’s a pretty like, regimented thing that you could teach an AI to do.

[Participant 6]

Symptom tracking and early detection were also identified as potential advantages. One participant described openness to AI-driven monitoring tools contingent upon clinician involvement while rejecting the idea of a fully AI therapist:

If I had an AI bot that was my therapist I don’t really know if I would like that, but…If I had maybe a piece of software on my phone that learned my habits…when I was starting to be manic and go crazy that would be very, very helpful.

[Participant 11]

These accounts illustrate that participants were open to AI when positioned as a transparent, clinician-supervised tool that enhances efficiency and monitoring, but expressed reluctance toward AI functioning independently or replacing human therapeutic relationships.

## Discussion

### Summary of Findings

This study explored the perspectives of individuals with OCD regarding the role of AI in health care using a qualitative thematic analysis framework. Several themes emerged: (1) inaccuracy and inefficacy of AI in health care, (2) the absence of clinical judgment and experiential reasoning in AI systems, (3) the potential loss of therapeutic alliance and human connection, (4) risks related to privacy, data security, and emotional variability, and (5) conditional acceptance of AI when used as a clinician-supervised tool that complements, rather than replaces, human care. Overall, participants favored a human-centered approach to AI integration in psychiatric treatment.

### Algorithmic Rigidity Versus Human Clinical Reasoning

Participants highlighted a distinction between algorithmic pattern recognition and human clinical reasoning. While participants acknowledged that AI may be capable of processing large amounts of information and generating predictions, many questioned whether such outputs constitute true clinical judgment. Although ML systems are highly effective at identifying statistical patterns [26], most lack clear mechanistic reasoning [27]. Participants recognized this limitation, expressing concern that AI-generated answers do not provide transparent reasoning or contextual interpretation. Their reflections align with models of diagnostic reasoning that involve both pattern recognition and analytical processes, which clinicians shift between intuitively [28]. In contrast, participants described psychiatric clinical decision-making as an interpretive and narrative process. Clinicians were viewed as integrating multiple forms of information – patient history, tone of speech, affect, and broader life context – to construct a meaningful understanding of an individual’s experience. Clinical literature describes this as a form of deliberate, analytical reasoning that extends beyond algorithmic classification and accounts for diagnostic uncertainty [29], and participants’ valuation of flexible reasoning in atypical cases reflects this same principle. Several individuals described experiences with unusual medication responses or highly individualized symptom presentations, emphasizing the need for clinicians to consider possibilities outside statistical norms [28]. Although participants in our study did not raise concerns about biases in human clinical decision-making, it is important to recognize that clinicians are also susceptible to cognitive biases and diagnostic errors. Emerging evidence suggests that generative AI models may exhibit less trauma-related diagnostic overshadowing bias than mental health professionals in certain structured diagnostic tasks, underscoring that both human and AI decision-making have distinct strengths and limitations [30].

OCD symptomatology may further amplify these concerns. Core features of OCD include intolerance of uncertainty and heightened distress in response to ambiguous or potentially threatening information [31]. Within this context, the possibility that AI could generate inaccurate or misleading outputs may be particularly distressing for individuals with OCD. As a result, perceived reliability and transparency may be especially important factors influencing acceptance of AI technologies in this population. Additionally, the prominent emphasis on reassurance and validation presented in Theme E should be interpreted within the context of this population, as reassurance seeking is a well-recognized feature of OCD and may therefore have shaped participants’ perspectives on the therapeutic role of human clinicians. Indeed, improvements in reassurance seeking track improvements in OCD [32] and excessive reassurance seeking can maintain OCD symptoms [33]. Increasingly, there is a recognition that the availability of generative AI to engage in the provision of reassurance may uniquely interact with OCD [34].

### Data Security, Trust, and Emotional Vulnerability Through the Lens of OCD

Another finding was the distinction participants drew between traditional privacy concerns and broader issues of emotional vulnerability when interacting with AI systems. Participants expressed concerns regarding the security of sensitive mental health data, including fears of data breaches, unauthorized access, or misuse of personal information. These concerns reflect well-documented challenges related to data governance and cybersecurity within digital health systems, including increasing rates of health care data breaches, hacking, malware attacks, and unauthorized access to sensitive health records [35].

Participants also described a second dimension of concern that extended beyond technical privacy protections and reported discomfort with disclosing deeply personal thoughts, fears, and compulsions to an AI system. For these participants, emotional vulnerability was not solely a matter of data protection but also of emotional trust and relational security, a distinction that has led to hesitance when disclosing sensitive information [36]. Technical safeguards such as encryption and regulatory protections may address risks related to data security, though may not resolve concerns related to emotional vulnerability or perceived impersonality. Participants frequently contrasted their trust in clinicians, who are bound by professional ethics and regulatory frameworks, with their uncertainty regarding how AI systems store, process, or use personal data, a hesitance documented across systematic reviews of patient attitudes toward AI [37,38].

OCD-related cognitive features may further influence these concerns. Individuals with OCD often demonstrate heightened sensitivity to issues of control, responsibility, and potential harm, as well as increased vigilance in response to perceived threats [39]. Uncertainty regarding how personal information might be used or shared in AI systems may therefore be particularly salient for this population. Participants’ emphasis on autonomy and transparency suggests that clear explanations of data storage, processing, and safeguards may be essential for fostering trust in AI mental health tools [40].

### The Irreplaceable Role of Therapeutic Alliance in Psychiatric Care

Participants consistently emphasized that certain components of psychiatric care are inherently human-centric–such as empathy, reassurance, emotional attunement, and individualized understanding–and therefore difficult to replicate through AI systems. These findings align with psychotherapy literature demonstrating that the therapeutic relationship between patient and clinician is one of the strongest predictors of treatment outcomes across therapeutic modalities [41,42].

In the context of OCD treatment, these relational factors may be especially important. ERP, the gold-standard psychotherapy for OCD, requires patients to confront anxiety-provoking stimuli while refraining from compulsive behaviors [43]. This process often generates significant distress, and successful engagement frequently depends on a strong therapeutic alliance and trust in the clinician guiding the exposure exercises. This is especially true of individuals with OCD of a more fearful or anxious attachment style [44]. Overall, participants described reassurance, trust, and emotional presence as critical components of effective OCD care, particularly during periods of symptom exacerbation.

Participants also emphasized that clinicians bring experiential knowledge to treatment through training, prior clinical encounters, and in some cases shared personal experiences, which they viewed as inherently difficult for AI systems to replicate. This act of “self-disclosure” by therapists has been previously shown to correlate with positive and therapeutic patient outcomes [45]. These perceptions suggest that psychiatric care may be viewed by patients as less amenable to automation than some other areas of medicine.

### Supported Roles of AI With Provider Supervision

While participants expressed concerns about AI’s role in psychiatric care, they did not outright reject the use of technology. Instead, participants expressed interest in AI systems that automate administrative tasks or provide decision support. For instance, participants described AI as potentially useful for intake screening, triage processes, and the organization of clinical information [46]. Automated collection of baseline information or standardized symptom assessments were viewed as ways to streamline patient care and clinical workflows. In this sense, AI was perceived as a tool that could improve efficiency without replacing clinician judgment. In turn, these systems could ultimately allow clinicians to devote more time to direct patient interaction [47].

Participants also expressed openness to AI-enabled symptom monitoring and clinical decision support, describing interest in tools that could track behavioral patterns, detect early changes in symptom severity, or assist clinicians in reviewing research evidence and evaluating treatment options. However, participants consistently emphasized that final clinical decisions should remain under human supervision. This perspective aligned with broader discussions that position AI as augmenting rather than replacing clinician expertise [48]. Importantly, participants’ conditional acceptance of AI appeared to reflect pragmatic openness rather than technological resistance. These findings suggest several practical considerations for the design of AI mental health tools. Developers could prioritize transparency, explainability, and clinician oversight when designing AI systems intended for psychiatric use. Safeguards for sensitive mental health data and mechanisms for personalization may also be important for addressing patient concerns and promoting trust.

### Limitations

This study has several limitations that should be considered when interpreting its findings. First, the participant sample skewed relatively young and was geographically concentrated in California, which likely reflects our recruitment methods. Participants were recruited both through in-person studies conducted in Los Angeles and through online OCD advocacy networks. The younger age distribution may partially reflect online recruitment strategies, as younger adults tend to spend more time online than older adults [49], and similar age distributions have been reported in other studies using online recruitment methods [50-52]. As with most qualitative research, the goal of this study was to explore experiences and perspectives in depth rather than to produce findings that are statistically generalizable.

Second, participation required a baseline level of technological familiarity, as individuals needed to access digital recruitment materials and complete interviews via Zoom. As a result, the perspectives of individuals with lower digital literacy, limited technology access, or significant discomfort using digital tools may be underrepresented. In addition, participants likely varied in their familiarity with different forms of AI, and many may have used the term “AI” broadly rather than distinguishing between LLMs, clinical decision support systems, predictive algorithms, or other AI-enabled technologies. While the interview guide contained one question eliciting opinions on physicians or therapists using AI to guide treatment, the topic of AI arose organically throughout interviews. Because of this, some responses may reflect different conceptualizations of AI. Accordingly, findings should be interpreted as participants’ perceptions of technologies they identified as AI rather than evaluations of any single AI modality.

Additionally, participants self-identified as having a diagnosis of OCD, and formal diagnostic assessments were not conducted. While self-reported diagnoses are commonly used in qualitative mental health research focused on lived experience and identity [53-56], some narratives may reflect overlapping symptoms with related conditions. To support credibility, research personnel reviewed transcripts for descriptions consistent with obsessive and compulsive experiences.

Relatedly, treatment history was not assessed. While diverse experiences may potentially impact attitudes toward the use of AI in treatment, prior research has found AI performance to be the biggest factor associated with trust of AI-assisted medical care, followed closely by the presence of a clinician [57].

An additional limitation is that the interview guide focused broadly on digital health technologies rather than AI specifically. While AI-related content was systematically identified across transcripts during coding, AI-focused questions represented only a small portion of the interview. Consequently, the themes identified should be interpreted as perspectives that emerged within a broader discussion of digital health technologies rather than as a comprehensive account of patient attitudes toward AI.

Finally, these findings should be interpreted as the perspectives of individuals living with OCD toward AI use in the context of mental health care. Some perspectives may generalize to broader populations and discussions of mental health care, while others may be more specific to individuals with OCD and their perspectives on OCD treatment.

Despite these limitations, this study provides novel insights into how individuals with OCD perceive the use of AI in health care and offers a foundation for future research examining patient-centered perspectives on emerging mental health technologies.

### Conclusions

Further research should examine patient acceptance of AI-enabled mental health tools using quantitative methods to assess uptake and usage patterns for specific applications such as symptom monitoring, intake triage, and clinical decision support systems. Comparative studies across psychiatric conditions may also clarify whether attitudes observed in individuals with OCD reflect broader populations or disorder-specific concerns.

Additional research is needed to evaluate the clinical effectiveness of AI-supported interventions for OCD, particularly tools designed to augment ERP through symptom monitoring, behavioral tracking, or between-session support. Recent work has supported this role for AI within exposure therapy [58]. Investigating how specific OCD symptom dimensions influence technology acceptance may also provide insight into which patient groups are most likely to benefit from AI-enabled care.

Finally, future work should explore ethical frameworks and governance models for the integration of AI into mental health care, with particular attention to transparency, clinician oversight, data security, and patient autonomy. Incorporating AI into mental health care has shown promise, though ongoing efforts to develop and implement a framework for ethical usage are needed [59,60]. Developing systems that prioritize explainability and protect sensitive mental health data may be essential for promoting trust and responsible adoption.

## Supplementary material

10.2196/98822 Multimedia Appendix 1 Demographic and training characteristics of the research team members and their involvement in conducting qualitative interviews.

10.2196/98822 Multimedia Appendix 2 Sample interview guide.

10.2196/98822 Multimedia Appendix 3 Codebook.

10.2196/98822 Checklist 1 COREQ checklist.

## Abbreviations

COREQ: Consolidated Criteria for Reporting Qualitative Research
*DSM-5*: *Diagnostic and Statistical Manual of Mental Disorders, 5th Edition*
ERP: exposure and response prevention
LLM: large language model
OCD: obsessive-compulsive disorder
PI: principal investigator
YBOCS: Yale Brown Obsessive Compulsive Scale
