# Supplementary material for: Perspectives of Individuals With Obsessive-Compulsive Disorder on the Role of Artificial Intelligence in Therapy and Treatment: Thematic Qualitative Study
Source: J Particip Med. 2026 Jul 31;18:e98822. doi: 10.2196/98822 (PMC13426896; doi:10.2196/98822)
Supplement: Multimedia Appendix 1 [file jopm-v18-e98822-s001.docx]

Research Team

Demographic and training characteristics of the research team members and their involvement in conducting qualitative interviews.

| Initials | Education/Occupation | Gender | Conducted Interviews |
| --- | --- | --- | --- |
| ACF | MD, PhD | Man | Yes |
| LO | Medical student | Non-binary | Yes |
| EL | Undergraduate student | Woman | Yes |
| KSP | Undergraduate student | Woman | Yes |
| TV | Medical student | Man | Yes |
| MM | Undergraduate student | Woman | Yes |
| TBAE | Undergraduate student | Man | Yes |
| ES | Undergraduate student | Woman | Yes |
| HW | Medical student | Man | No |
| DM | Medical student | Man | No |
| EG | Research Assistant | Woman | No |
| DS | Medical student | Man | No |
