# Supplementary material for: Perspectives of Individuals With Obsessive-Compulsive Disorder on the Role of Artificial Intelligence in Therapy and Treatment: Thematic Qualitative Study
Source: J Particip Med. 2026 Jul 31;18:e98822. doi: 10.2196/98822 (PMC13426896; doi:10.2196/98822)
Supplement: Multimedia Appendix 3 [file jopm-v18-e98822-s003.docx]

Codebook

Global Codes

**New Finding**

- For data that is important to our research questions, or is interesting, but is not captured by current codes

**Good Quote**

**Parent Code**
**Child Code**

- **Concern:** Describing, discussing, or referencing an aspect of AI as concretely or potentially concerning, negative, dangerous, etc
  - **Bias:** References to the potential of AI to increase or add-to biases in healthcare, such as those related to race, gender, socioeconomic status, or other personal characteristics.
  - **Efficacy:** Any mention of concerns regarding the effectiveness of AI-guided treatment. This may include doubts about whether AI can deliver outcomes comparable to human-led therapy, skepticism about the adequacy of AI in addressing complex or nuanced mental health issues, or apprehension that AI may not effectively tailor treatments to individual needs.
  - **Accuracy:** References to worries about the precision and correctness of AI’s recommendations, diagnoses, or treatment plans. This could involve concerns about the reliability of data used to train AI models, the potential for errors or misinterpretations in clinical settings, or the risk of AI generating inaccurate conclusions due to data quality issues or algorithm limitations.
  - **Autonomy:** Descriptions of fears related to the potential loss of patient or provider autonomy in treatment decisions due to AI involvement, and/or desiring adequate or additional disclosure and education of its uses.
  - **De-individualization:** Any concern that AI may lead to a more generalized, one-size-fits-all approach to therapy, potentially neglecting the unique aspects of individual patients' experiences, personalities, or treatment needs. This code captures worries that AI could promote standardized protocols at the expense of personalized care and empathy that human providers typically offer.
  - **Communication**: Any reference to concerns of communications developed or delivered from AI technology.
  - **Data Security:** Any reference to concerns of insufficient protection of private or sensitive information from unauthorized access and exfiltration
- **Benefit:** Describing, discussing, or referencing an aspect of AI as concretely or potentially beneficial, positive, etc.
  - **Standardization:** Any mention of the benefits associated with AI’s ability to provide consistent, uniform treatment recommendations, assessments, or interventions across different therapists or sessions, reducing variability due to human factors.
  - **Efficacy:** Any belief regarding the noninferiority or effectiveness of AI-guided compared to human-led treatment.
  - **Accuracy:** References to trust in the precision and correctness of AI’s recommendations, diagnoses, or treatment plans.
  - **Bias Reduction:** References to the potential of AI to reduce or eliminate biases in healthcare, such as those related to race, gender, socioeconomic status, or other personal characteristics, thereby promoting fairer and more equitable care.
  - **Communication:** Any reference to benefits from communications developed or delivered from AI technology.
  - **Symptom Tracking:** Descriptions of how AI can aid in tracking and monitoring patient symptoms over time, offering real-time updates, predictive insights, and potentially improving the timeliness and accuracy of treatment adjustments based on objective data.
- **Complementary Use of AI:** refers to the perception that artificial intelligence should function as a supportive tool that enhances, rather than replaces, traditional mental health care.
